# Respiratory viral infections and the risk of rheumatoid arthritis

**DOI:** 10.1186/s13075-019-1977-9

**Published:** 2019-08-30

**Authors:** Young Bin Joo, Youn-Hee Lim, Ki-Jo Kim, Kyung-Su Park, Yune-Jung Park

**Affiliations:** 1 Division of Rheumatology, Department of Internal Medicine, St. Vincent’s Hospital, College of Medicine, The Catholic University of Korea, Seoul, Republic of Korea; 2 Institute of Environmental Medicine, Seoul National University Medical Research Center, Seoul, Republic of Korea; 3 Environmental Health Center, Seoul National University College of Medicine, Seoul, Republic of Korea

**Keywords:** Respiratory viral infections, Rheumatoid arthritis, Risk factor

## Abstract

**Background:**

We aimed to investigate the effects of ambient respiratory viral infections in the general population on rheumatoid arthritis (RA) development.

**Methods:**

Data of weekly incident RA (2012–2013) were obtained from the Korean National Health Insurance claims database, and those of weekly observations on eight respiratory viral infections were obtained from the Korea Centers for Disease Control and Prevention database. We estimated the percentage change in incident RA associated with ambient mean respiratory viral infections using a generalized linear model, after adjusting for time trend, air pollution, and meteorological data.

**Results:**

A total of 24,117 cases of incident RA (mean age 54.7 years, 18,688 [77.5%] women) were analyzed. Ambient respiratory viral infections in the population were associated with a higher number of incident RA over time, and its effect peaked 6 or 7 weeks after exposure. Among the 8 viruses, parainfluenza virus (4.8% for 1% respiratory viral infection increase, 95% CI 1.6 to 8.1, *P* = .003), coronavirus (9.2%, 3.9 to 14.8, *P* < .001), and metapneumovirus (44%, 2.0 to 103.4, *P* = .038) were associated with increased number of incident RA. The impact of these respiratory viral infections remained significant in women (3.8%, 12.1%, and 67.4%, respectively, *P* < .05) and in older patients (10.7%, 14.6%, and 118.2%, respectively, *P* < .05).

**Conclusions:**

Ambient respiratory viral infections in the population were associated with an increased number of incident RA, especially in women and older patients, suggesting that respiratory viral infections can be a novel environmental risk factor for the development of RA.

**Electronic supplementary material:**

The online version of this article (10.1186/s13075-019-1977-9) contains supplementary material, which is available to authorized users.

## Introduction

Rheumatoid arthritis (RA) is an immune-mediated disease involving interactions between genetic and environmental factors [1, 2]. It has been considered that a pre-clinical RA phase comprising the generation of autoantibodies in genetically susceptible individuals lasts months to years then transitions to a clinical RA event by virtue of other driving factors [2]. These driving factors are currently poorly understood, but it is suspected that microvascular, neuroregulatory, microtrauma-related, or transient infection-dependent pathways are involved [2, 3].

We are interested in determining whether respiratory viral infections have the capacity to driving RA development, for several reasons. Smoking, periodontitis, and microbiomes—all prominent environmental risk factors for RA—interact with mucosal surfaces including the lungs, oral mucosa, and gastrointestinal tract [4]. It is thought that these local tissue stresses on the mucosa lead to post-translational modification of peptides involved in RA pathogenesis [2, 5, 6]. Initial respiratory virus infections usually involve both the oral mucosa and the lungs, and this may be relevant to the generation of immune responses potentially associated with RA development. Previous studies suggest that RA exhibits seasonal tendencies, whereby RA onset is more frequent in winter [7], and relapses are more frequent in summer [8]. Thus, we hypothesized that respiratory viral infections that exhibit seasonality may be associated with RA development. This hypothesis is supported by studies investigating other autoimmune diseases [9–17]. Multiple sclerosis which exhibits seasonal tendencies has been associated with upper respiratory picornavirus, rhinovirus, and influenza infections [9–15]. Influenza virus infections triggered disease in a genetic model of experimental autoimmune encephalomyelitis [16]. Furthermore, the occurrence of pediatric Henoch-Schönlein purpura was highest in the spring and lowest in the summer, and it was associated with an outbreak of influenza [17].

A few studies have investigated a potential link between respiratory viral infections and the development of RA [18, 19]. In the population-based case-control study, previous respiratory tract infections including sinusitis and tonsillitis treated with antibiotics, and pneumonia showed no association with a risk of RA [18]. In this study, it is not certain whether respiratory infections were caused by viruses or bacteria, but rather bacterial respiratory infections seemed to be considered more. Another study, however, showed that viral infection symptoms confirmed by questionnaire were more frequent in patients with a new-onset RA in the previous year compared to healthy control, but this was a small-sized study using 59 RA patients and 69 controls [19].

Reliable data reflecting respiratory viral epidemic burden in South Korea are available, because the Korea Centers for Disease Control and Prevention (KCDC) operates a well-established surveillance system for the detection of respiratory viruses via polymerase chain reaction (PCR) diagnosis in patients with respiratory symptoms [20, 21]. In addition, population-based incident RA can be identified via claims data because the Korean National Health Insurance (KNHI) covers almost the entire South Korean population.

In the present study, the effects of ambient respiratory viral infections on the number of incident RA in South Korea were investigated using national public data. First, patterns of incident RA over time were investigated using claims data. A time-series analysis was then conducted to investigate associations between the detection rate of ambient respiratory viral infections and the number of incident RA.

## Methods

### Study design and data source

This is an ecological study design, and we used records from the KNHI claims database from 2011 to 2015. Patients’ diagnoses recorded via the International Statistical Classification of Diseases and Related Health Problems 10th Revision (ICD-10), procedures, prescriptions, type of institution or department, and individual beneficiary information were provided [22]. The protocol utilized in the present study was approved by the Institutional Ethics Review Board of St. Vincent’s Hospital, Catholic University of Korea.

### Incident RA

The algorithm for identifying RA using claims has been previously validated in Korea [23] and was recently updated by Won et al. [24]. In accordance with Won et al. [24], we selected individuals aged ≥ 19 years with claims data pertaining to RA (ICD-10 codes M05 or M06). RA was deemed to be confirmed in cases in which a prescription for disease-modifying antirheumatic drugs was issued within 1 year of the RA code being assigned. Incident RA, which means new RA cases, has to be fulfilled 1-year window period (no codes or prescriptions for RA) and three consecutive years of treatment. Weekly number of incident RA was calculated from the first week of January 2012 to the last week of December 2013.

### Respiratory virus data

KCDC posts the incidences of respiratory virus infections each week on their website (http://cdc.go.kr) [20]. Nasopharyngeal specimens from patients with acute respiratory infections are collected from 36 sentinel hospitals located nationwide and subjected to respiratory viral genetic testing via multiplex PCR. Target viruses include influenza, parainfluenza, adenovirus, respiratory syncytial virus (RSV), rhinovirus, coronavirus, metapneumovirus, and bocavirus.

The detection rate of respiratory virus was calculated as a proportion of patients who are confirmed for viral infection by PCR among those with acute respiratory viral infection symptoms who visited sentinel hospitals. Because we hypothesized that respiratory viral infections would exhibit a delayed association with incident RA rather than an immediate effect, the detection rates of eight respiratory viruses were collected from the first week of November 2011, which is 8 weeks prior to the start date of the collection of incident RA data, to the last week of December 2013.

### Environmental factors as potential confounders

Data pertaining to the potentially confounding factors with regard to the viral detection rates and RA diagnoses were obtained from public websites. We obtained hourly air pollution data including particulate matter < 10 μg/m^3^ in aerodynamic diameter (PM_10_) and ozone (O_3_) from the website airkorea.gov.kr [25], operated by the Korean Ministry of Environment. Meteorological data reflecting hourly measurements of temperature, humidity, and solar radiation were obtained from the website maintained by the Korea Meteorological Administration [26]. The hourly mean of all variables was calculated by using obtained raw data in each station and converted to daily means. Next, the daily metrological data were converted into weakly means, then these means were analyzed in conjunction with the respiratory viral infection data. As with the respiratory viral detection rate data, the meteorological data were collected from the first week of November 2011 to the last week of December 2013.

### Subgroup analysis

To identify the groups who were significant to the effects of ambient respiratory viral infections on the number of incident RA, a subgroup analysis was conducted based on age, sex, and the presence or absence of respiratory disease prior to RA development. Age groups were categorized as < 40 years, 40–59 years, and ≥ 60 years based on previously reported definitions of young-onset and elderly-onset RA [27]. The presence of respiratory diseases was defined as cases with respiratory disease codes during the 12 months prior to RA diagnosis. Respiratory disease codes were extracted from ICD-10 codes (I27.8, I27.9, J40.x-J47.x, J60.x-J67.x, J68.4, J70.1, J70.3) for Charlson comorbidity index analysis [28].

### Statistical analysis

Because respiratory virus data are provided by the source as nationwide totals, all other data were analyzed as nationwide totals. First, generalized additive modeling (GAM) with integrated smoothness estimation was used to investigate the relationships between detection rates of eight respiratory viruses and the numbers of incident RA cases. Generalized linear modeling (GLM) was then used to estimate the effects of eight respiratory viruses on the numbers of incident RA cases after adjusting for potential confounders.

Degrees of freedom (*df*) for each confounding factor was determined based on the unbiased risk estimation derived from the GAM. Potential confounders used in the model were PM_10_ with 9 *df*, O_3_ with 9 *df*, mean temperature with 8 *df*, mean humidity with 9 *df*, solar radiation with 9 *df*, and the natural cubic splines (ns) of time trend with 4 *df* per year (4 *df* × 2 years = 8 *df*). To consider delayed and cumulative effects of respiratory viral infections on incident RA, we used the moving average lag up to eight lag weeks (lag1–8). For example, “lag1–8” refers to a moving average lag model for respiratory viral infections over the previous 8 weeks. Confounder lag weeks were also matched with those of each virus in GLM. To determine the greatest respiratory viral effect on incident RA, we selected the lag associated with the highest beta for each virus then analyzed the statistical significance of the effect size at the selected lag week in each virus.

SAS statistical software (version 9.4, SAS, Cary, NC, USA) was used for data collation. All statistical analyses were performed using R software (version 3.5.1, The R Project for Statistical Computing, www.r-project.org). A *P* value < .05 was considered statistically significant.

## Results

### Baseline characteristics

From January 2012 to December 2013, the total number of patients newly diagnosed with RA was 24,117. Of these, 18,688 (77.5%) were females, and the mean age at RA diagnosis was 54.7 (SD 13.2) years (Table 1). Almost all patients (95.0%) had a national health insurance. The proportion of institutions that reported patients who were diagnosed with a new RA was 44.6%, 23.8%, and 31.6% in clinics, general hospitals, and tertiary hospitals, respectively (Additional file 1: Table S1).

**Table 1:** Characteristics of patients newly diagnosed with rheumatoid arthritis in 2012 or 2013

Clinical values	Patients (*n* = 24,117)
Age	
Overall mean ± SD (years)	54.7 ± 13.2
Age group frequencies (*n* and %)	
19–39 years	3174 (13.2%)
40–59 years	12,332 (51.1%)
≥ 60 years	8611 (35.7%)
Sex (*n* and %)	
Women	18,688 (77.5%)
Men	5429 (22.5%)
Previous respiratory diseases (*n* and %)	
Absence	14,273 (59.18%)
Presence	9844 (40.82%)
Number of patients per year (*n* and %)	
2012	12,024
2013	12,093

*SD* standard deviation

### Seasonal tendency of incident RA

**Fig. 1:**
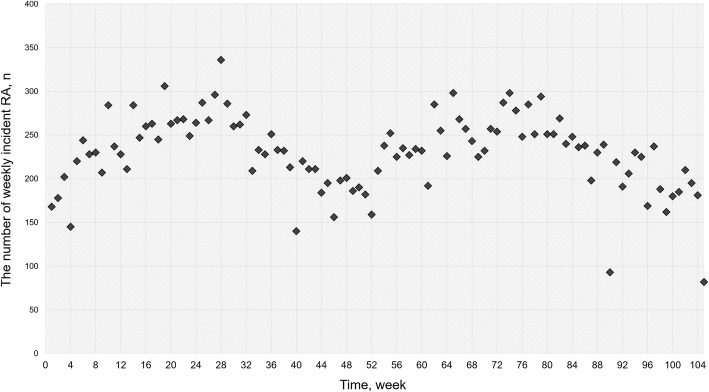
Incident rheumatoid arthritis trends in 2012 and 2013. The *x*-axis represents the study period from 2012 to 2013. Because respiratory virus data (exposure) are provided weekly, the number of incident RA cases (outcome) is also represented weekly from the first week of January 2012 to the last week of December 2013. The *y*-axis represents the weekly number of incident RA cases in each week

The analysis revealed a seasonal tendency of incident RA in each year (Fig. 1). In 2012, the number of incident RA increased from January (*n* = 781) to July (*n* = 1274) then decreased to December (*n* = 775) (Additional file 1: Table S2). July had the highest number of incident RA cases, and December had the lowest. We observed a similar seasonality in 2013. In that year, the number of incident RA cases increased from January (*n* = 1057) to July (*n* = 1212) then decreased to December (*n* = 853). The differences in the weekly mean number of incident RA cases were compared between the top peaks in July and other months. A significantly lower number of weekly incident RA cases were observed from September to February in both 2012 and 2013 (Additional file 1: Table S2). Men and women showed the same seasonal pattern for the number of incident RA (Additional file 1: Figure S1).

### Effects of respiratory viral infections on incident RA

Most viruses showed seasonal tendencies between 2012 and 2013 (Additional file 1: Figure S2). Outbreaks of parainfluenza virus and bocavirus tended to occur in late spring and early summer. RSV tended to be diagnosed in the autumn. Outbreaks of influenza occurred in winter, and coronavirus tended to be diagnosed from November to December. Rhinovirus was prevalent from spring to autumn, but only exhibited an outbreak in the autumn of 2012, not in the autumn of 2013. Metapneumovirus was infrequent but tended to be diagnosed in late winter and spring. Adenovirus did not exhibit any seasonal tendencies.

The associations between the detection rate of respiratory viral infections and the number of incident RA were investigated using GAM. At lag1w, fluctuations in associations were observed for all viruses except bocavirus (Additional file 1: Figure S3). As the lag structure changed from lag1w to lag1–8w, however, the fluctuations in the associations gradually disappeared and the association became more apparent and more linear for some viruses. This suggests that ambient respiratory viral infections are associated with an increased number of incident RA after a moderate time interval, rather than having an immediate effect.

**Fig. 2:**
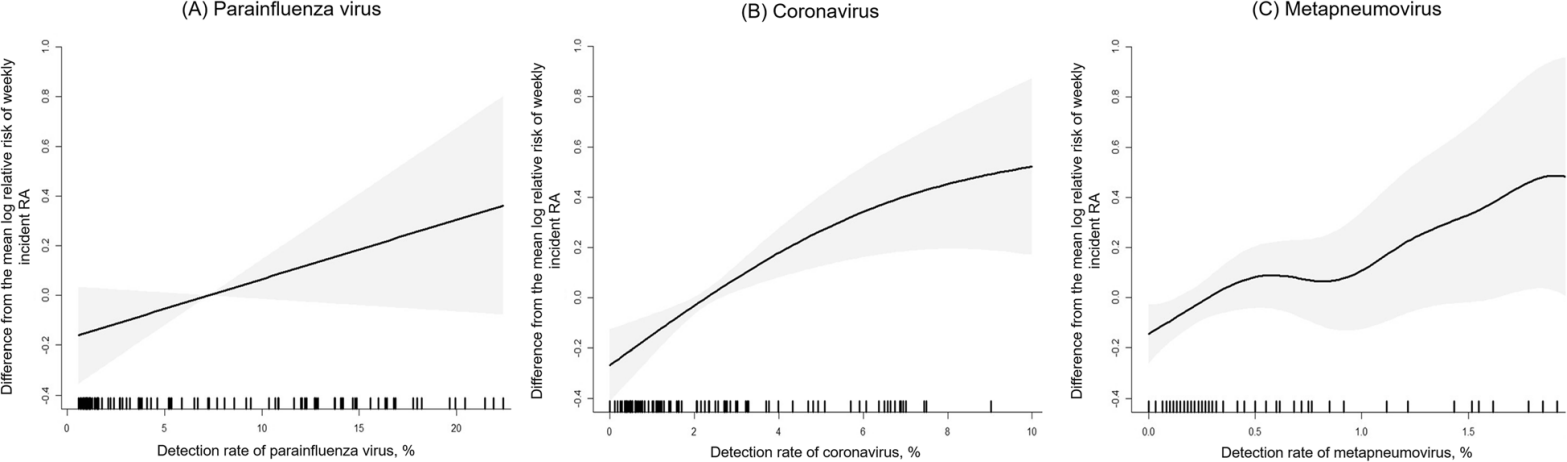
Effects of ambient respiratory viral infections on incident RA. Parainfluenza (**a**), coronavirus (**b**), and metapneumovirus (**c**) infection. The *x*- and *y*-axes represent the weekly virus detection rate as a percentage and difference from the mean log relative risk of incident RA, respectively. Solid lines represent the associations between the weekly virus detection rate and weekly number of incident RA cases, and the gray area represents 95% confidence intervals for the risk

Three viruses were significantly associated with the number of incident RA in time-series analysis (Table 2). The number of incident RA increased by 4.8% with an incremental increase of 1% in the parainfluenza virus detection rate at lag1–7w (*P* = 0.003). At lag1–6w, coronavirus and metapneumovirus were associated with respective increases in the number of incident RA of 9.2% (*P* < .001) and 44.0% (*P* = 0.038) with an incremental increase of 1% in virus detection rate. The visualized associations with integrated smoothness estimation of these respiratory viruses are shown in Fig. 2a–c.

**Table 2:** The effects of ambient respiratory virus on incident RA

Virus	Percent change in incident RA per 1-unit change in the detection rate of virus^a^	95% CI	*P* value
Adenovirus at lag1–6w	1.93	− 1.14 to 5.09	.220
Parainfluenza virus at lag1–7w	4.80	1.57 to 8.14	.003
Respiratory syncytial virus at lag1w	− 0.06	− 0.82 to 0.71	.873
Influenza virus at lag1–6w	0.42	− 0.68 to 1.53	.446
Corona virus at lag1–6w	9.20	3.85 to 14.82	< .001
Rhinovirus at lag1–8w	1.54	− 2.19 to 5.42	.423
Bocavirus at lag1–8w	13.33	− 1.57 to 30.48	.082
Metapneumovirus at lag1–6w	44.02	1.98 to 103.39	.038

The moving average lag up to 8 lag weeks (lag1–8) was used in the analysis. For example, “lag1–8” refers to a moving average lag model for respiratory viral infections over the previous 8 weeks

*CI* confidence interval

^a^Relative risk adjusted for mean particulate matter < 10 μg/m^3^ in aerodynamic diameter, ozone, temperature, humidity, amount of solar exposure, and seasonality

### Subgroup analysis

In women, all three viruses were significantly associated with incident RA (*P* < .05), but in men, only parainfluenza virus was (*P* = 0.019) (Table 3). Elderly-onset RA in patients aged ≥ 60 years was significantly associated with all three viruses (*P* < .05). Only parainfluenza virus was significantly associated with incident RA in patients aged under 40 years (*P* = 0.034), and in patients aged 40–59 years, none of the three viruses was significantly associated with incident RA. The presence or absence of preexisting respiratory diseases showed a different association with each virus; that is, coronavirus was only associated with the number of incident RA cases in the absence of other respiratory conditions, while metapneumovirus showed the opposite results, i.e., a significant association (79.18% change in risk per 1-unit change in exposure, *P* = 0.034) in the presence of a respiratory disease, but not in disease absence (23.87% change in risk per 1-unit change in exposure, *P* = 0.352).

**Table 3:** The effects of ambient respiratory virus on incident RA in subgroups of patients

Subgroup	Percent change in incident RA per 1-unit change in the detection rate of virus^a^	95% CI	*P* value
Sex			
Men			
Parainfluenza virus at lag1–7w	8.24	1.34, 15.61	.019
Corona virus at lag1–6w	0.20	− 9.77, 11.28	.971
Metapneumovirus at lag1–6w	− 5.11	− 58.96, 75.59	.659
Women			
Parainfluenza virus at lag1–7w	3.82	0.18, 7.59	.040
Corona virus at lag1–6w	12.11	5.87, 18.71	< .001
Metapneumovirus at lag1–6w	67.4	13.04, 147.88	< .001
Age			
< 40 years			
Parainfluenza virus at lag1–7w	10.02	0.73, 20.17	.034
Corona virus at lag1–6w	7.97	− 6.31, 24.43	.289
Metapneumovirus at lag1–6w	45.89	− 43.27, 275.18	.433
40–59 years			
Parainfluenza virus at lag1–7w	− 0.11	− 4.38, 4.35	.962
Corona virus at lag1–6w	5.64	− 1.50, 13.3	.124
Metapneumovirus at lag1–6w	5.52	− 34.93, 71.09	.828
≥ 60 years			
Parainfluenza virus at lag1–7w	10.66	4.96, 16.67	< .001
Corona virus at lag1–6w	14.64	5.41, 24.67	.001
Metapneumovirus at lag1–6w	118.19	22.19, 289.61	.008
Previous respiratory disease			
Without respiratory disease			
Parainfluenza virus at lag1–7w	4.48	0.26, 8.87	.037
Corona virus at lag1–6w	10.61	3.58, 18.11	.003
Metapneumovirus at lag1–6w	23.87	− 21.09, 94.47	.352
With respiratory disease			
Parainfluenza virus at lag1–7w	5.66	0.61, 10.97	.028
Corona virus at lag1–6w	6.72	− 1.43, 15.53	.108
Metapneumovirus at lag1–6w	79.18	4.52, 207.16	.034

The moving average lag up to 8 lag weeks (lag1–8) was used in the analysis. For example, “lag1–8” refers to a moving average lag model for respiratory viral infections over the previous 8 weeks

*CI* confidence interval

^a^Relative risk adjusted for mean particulate matter < 10 μg/m^3^ in aerodynamic diameter, ozone, temperature, humidity, amount of solar exposure, and seasonality

## Discussion

The idea of infections acting as a trigger for the development of RA has been suggested for a quite long time without much clarification. In this study, the number of weekly incident RA cases exhibited an inverted U-shaped seasonal tendency throughout each year. In addition, the detection rate for ambient respiratory viral infections in the population was associated with an increased number of incident RA cases, which suggests a possible role for respiratory infections as a trigger for the development of RA.

Seasonal tendencies of RA onset or relapse have been evaluated in a small number of studies. In an Italian study including 44 RA patients, there was no seasonal tendency [29]. In the UK between 1957 and 1963, 43 of the 100 patients had reported that RA occurred during the winter [7]. In a more recent study using data from the year 2000 in Israel, RA relapse occurred mostly during the summer [8]. These studies were conducted many years ago with small study groups. Advances in the understanding of RA and in diagnostic tools have since changed the reported characteristics of patients with RA. Thus, there is a need to reevaluate seasonal patterns of RA using recent data derived from large groups.

In the present study, population-based incident RA diagnoses in 2012 and 2013 were used, which is more reliable. Notably, however, observations pertaining to the seasonality of RA in the study should be interpreted with caution. The index date of incident RA is not the date of onset, rather it is the date that RA is diagnosed by physicians. The onset of RA may precede its diagnosis by several weeks or months [30]. Nevertheless, a distinct seasonal pattern of incident RA determined based on the date of diagnosis could be explained by the role of respiratory viral infections in inflammation. Respiratory viral infections may play a role in the exacerbation of inflammation involving joints in patients with subclinical or early-stage RA, prompting patients to visit the hospital.

Several mechanisms of virus-induced initiating or triggering of autoimmune disease have been suggested [31, 32]: (1) “Molecular mimicry” is the most widely proposed mechanism and occurs when a virus antigen mimics a host antigen and activates cross-reactive T cells. (2) “Epitope spreading” is another potential mechanism. Tissue damage resulting from virus-specific T cell activation or direct virus-mediated host tissue destruction causes de novo activation of autoreactive T cells and releases self-antigens into the inflammatory environment. (3) “Bystander activation” is the activation of autoreactive T cells as a result of the release of cytokines during a virus-targeted immune response. (4) Encrypted host antigens are released from certain tissues during virus-targeted tissue damage. (5) “Superantigens” activate a wide range of nonspecific T cell clones regardless of their specificity. In this study, however, we could only reveal the association between respiratory viruses and incident RA, but not investigate the possible mechanisms as this is an ecological study.

In our study, parainfluenza, coronavirus, and metapneumovirus were significantly associated with the number of incident RA. The patterns of virus seasonality, the severity or virulence of infections, and the parameters such as peak age of viral infections have differential effects on the associations between each respiratory virus and incident RA. The similarity of the three significant respiratory viruses in the study has not been elucidated by one of the factors described above. More complex interactions among these virus factors or other environmental factors may be involved.

Regarding the confounding factors, we adjusted for meteorological factors and air pollution data, which could affect the seasonality of the number of incident RA cases. Considering the unmeasured confounders associated with seasonality, we also adjusted the time trend in the generalized linear model to avoid overestimation of the effects of respiratory viral infection on the number of incident RA cases. Nevertheless, other factors that could explain why the number of incident RA cases increased in July could exist. This point indicates we should be cautious in interpreting these results.

It was interesting that the effects of ambient respiratory viral infections in the population on the number of incident RA differed according to sex, although men and women showed the same seasonal pattern for the number of incident RA cases. Only the parainfluenza virus was significant in men while all three viruses were significant in women. The relatively small sample size of men might have affected this difference. Also, a stronger immune response in women could affect this difference [33]. Stronger immunity to pathogens in women is associated with lower viral loads and lower prevalence of infections than in men, but it may also be associated with increased severity of disease symptoms [34]. For example, HIV-positive women tend to exhibit less circulating HIV RNA than HIV-positive men, but they are reportedly at a 1.6-fold higher risk of developing AIDS [35]. Fatality following exposure to pathogenic influenza A viruses is higher in women [36, 37]. It may be that respiratory viral infections result in stronger inflammation in women than in men, and trigger stronger immune responses, and this results in a differential proportion of incident RA in women and men. However, the virus data in relation to sex was not available for analysis in the present study.

Viral arthritis is distinct from autoimmune disease-associated polyarthritis. Viral arthritis is usually self-limiting, and treatment with immunosuppressants is usually not required [38]. To exclude viral arthritis in the present study, only individuals who underwent treatment for 3 years were included. Additionally, treatment was required to include immunosuppressants. Thus, the seasonal tendency of RA diagnosis and the association between ambient respiratory viral infections in the population and incident RA in the study are unlikely to be an effect of transient viral arthritis.

The current study had some limitations. A causal association between ambient respiratory viral infections in the population and incident RA could not be proven because this is an ecological study where it is not known whether the individuals with RA also had viral infections. A clear causal association between respiratory viral infections and RA development remains to be proven via future individual-level data study. In addition, many respiratory viral infections, especially in the case of mild symptoms, would not have been detected at hospitals, hence would not have been listed in the nationwide database. Further study including all the patients with acute respiratory infection symptoms, not based on PCR, confirmed infection would also be valuable when investigating possible associations between respiratory infections and RA development.

## Conclusions

In the present study, RA development exhibited a seasonal tendency, and ambient parainfluenza, coronavirus, and metapneumovirus infections were associated with an increased number of incident RA. These results support the etiological hypothesis that respiratory viral infections in the population may have the capacity to trigger RA.

## Additional file

Additional file 1: **Table S1.** Payer type and institutions of patients newly diagnosed with rheumatoid arthritis in 2012 or 2013. **Table S2.** Monthly and weekly number of Incident RA. **Figure S1.** Incident rheumatoid arthritis trends according to sex in 2012 and 2013. **Figure S2.** The weekly detection rate of eight respiratory virus in 2012 and 2013. **Figure S3.** Risks of incident RA associated with infection with eight respiratory viruses over 8 lag weeks. (DOCX 1539 kb)

## Data Availability

The datasets generated and/or analyzed during the current study are available in the KNHI and KCDC at the KCDC website (http://www.cdc.go.kr/CDC/info/CdcKrInfo0502.jsp?menuIds=HOME006-MNU3003-MNU2953, available in Korean).

## Abbreviations

*df*: Degrees of freedom
GAM: Generalized additive modeling
GLM: Generalized linear modeling
ICD-10: International Statistical Classification of Diseases and Related Health Problems 10th Revision
KCDC: Korea Centers for Disease Control and Prevention
KNHI: Korean National Health Insurance
ns: Natural cubic splines
O_3_: Ozone
PCR: Polymerase chain reaction
PM_10_: Particulate matter < 10 μg/m^3^ in aerodynamic diameter
RA: Rheumatoid arthritis
RSV: Respiratory syncytial virus
