# Supplementary material for: Respiratory viral infections and the risk of rheumatoid arthritis
Source: Arthritis Res Ther. 2019 Aug 30;21:199. doi: 10.1186/s13075-019-1977-9 (PMC6716891; doi:10.1186/s13075-019-1977-9)
Supplement: Supplementary file 1 — Table S1. Payer type and institutions of patients newly diagnosed with rheumatoid arthritis in 2012 or 2013. Table S2. Monthly and weekly number of Incident RA. Figure S1. Incident rheumatoid arthritis trends according to sex in 2012 and 2013. Figure S2. The weekly detection rate of eight respiratory virus in 2012 and 2013. Figure S3. Risks of incident RA associated with infection with eight respiratory viruses over 8 lag weeks. (DOCX 1539 kb) [file 13075_2019_1977_MOESM1_ESM.docx]

**Table S1.** Payer type and institutions of patients newly diagnosed with rheumatoid arthritis in 2012 or 2013.

| Clinical values | Patients (*n* = 24,117) |
| --- | --- |
| Payer type (*n* and %) |  |
| National Health Insurance | 22,922 (95.0%) |
| Medicaid | 1,195 (4.7%) |
| Government-sponsored | 70 (0.3%) |
| Type of institutions (*n* and %) |  |
| Tertiary hospital | 7,630 (31.6%) |
| General hospital | 5,746 (23.8%) |
| Clinic and community hospital | 10,741 (44.6%) |

**Table S2.** Monthly and weekly number of Incident RA

|  | total | Weekly mean ± SD | P value* |
| --- | --- | --- | --- |
| In 2012 | 12,024 |  |  |
| January | 781 | 182.6 ± 29.3 | **0.0006** |
| February | 966 | 227.3 ± 15.3 | **0.0092** |
| March | 1,035 | 240.0 ± 31.3 | 0.0548 |
| April | 1,104 | 259.8 ± 15.6 | 0.1065 |
| May | 1,233 | 276.0 ± 20.1 | 0.5266 |
| June | 1,133 | 266.8 ± 15.6 | 0.2548 |
| July | 1,274 | 288.0 ± 31.0 | - |
| August | 1,067 | 235.8 ± 26.9 | **0.0325** |
| September | 953 | 213.8 ± 43.4 | **0.0144** |
| October | 889 | 206.5 ± 15.6 | **0.0021** |
| November | 814 | 187.5 ± 21.1 | **0.0009** |
| December | 775 | 179.3 ± 13.9 | **0.0003** |
| In 2013 | 12,093 |  |  |
| January | 1,057 | 231.8 ± 16.0 | **0.0393** |
| February | 919 | 221.3 ± 19.7 | **0.0236** |
| March | 1,088 | 266.4 ± 27.9 | 0.7391 |
| April | 1,091 | 239.3 ± 14.0 | 0.1220 |
| May | 1,211 | 274.0 ± 21.9 | 0.3968 |
| June | 1,080 | 265.5 ± 18.7 | 0.7492 |
| July | 1,212 | 261.0 ± 21.2 | - |
| August | 1,013 | 230.0 ± 22.0 | 0.0689 |
| September | 828 | 194.4 ± 59.5 | **0.0461** |
| October | 924 | 207.5 ± 27.7 | **0.0131** |
| November | 817 | 191.8 ± 32.1 | **0.0058** |
| December | 853 | 170.6 ± 50.8 | **0.0063** |

*The differences in weekly mean number of incident RA cases between the “top peaks” in July (reference) and other months were established by independent Student’s t-test.

*[Women]*


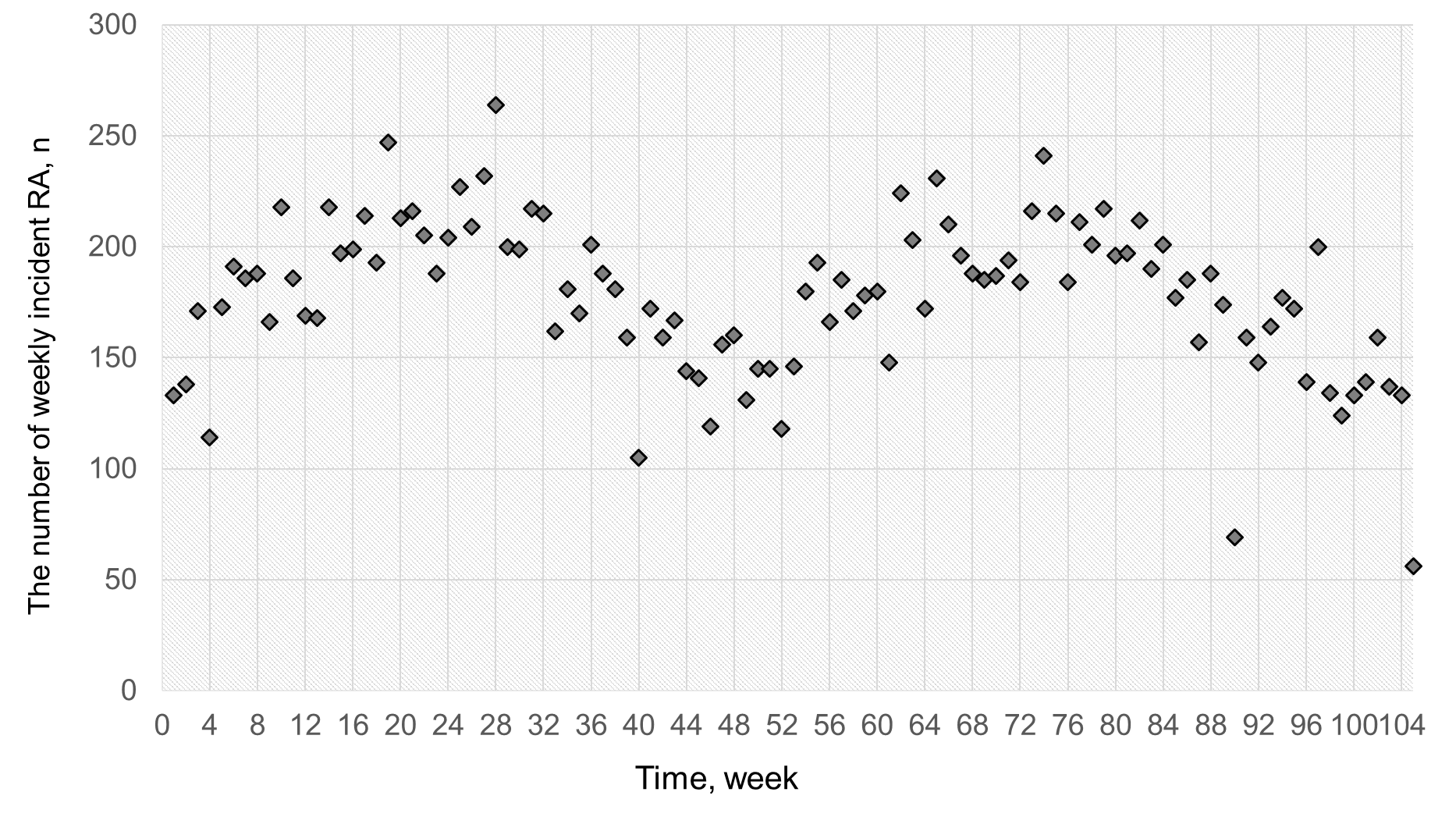


*[Men]*


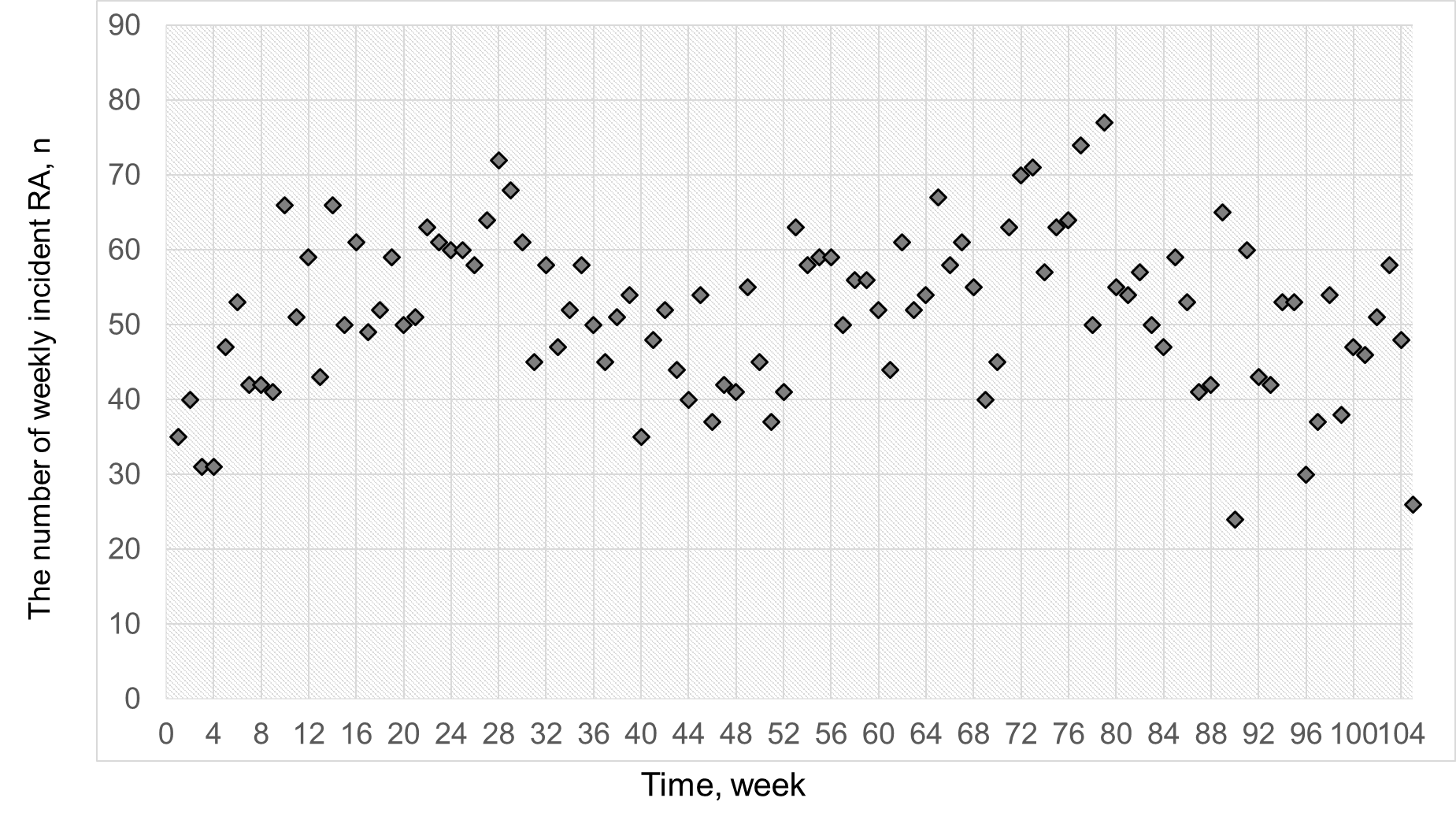


**Figure S1.** Incident rheumatoid arthritis trends according to sex in 2012 and 2013.

X-axis represent the time of study period from 2012 to 2013. Y-axis represent the monthly number of incident RA.

[Adenovirus]


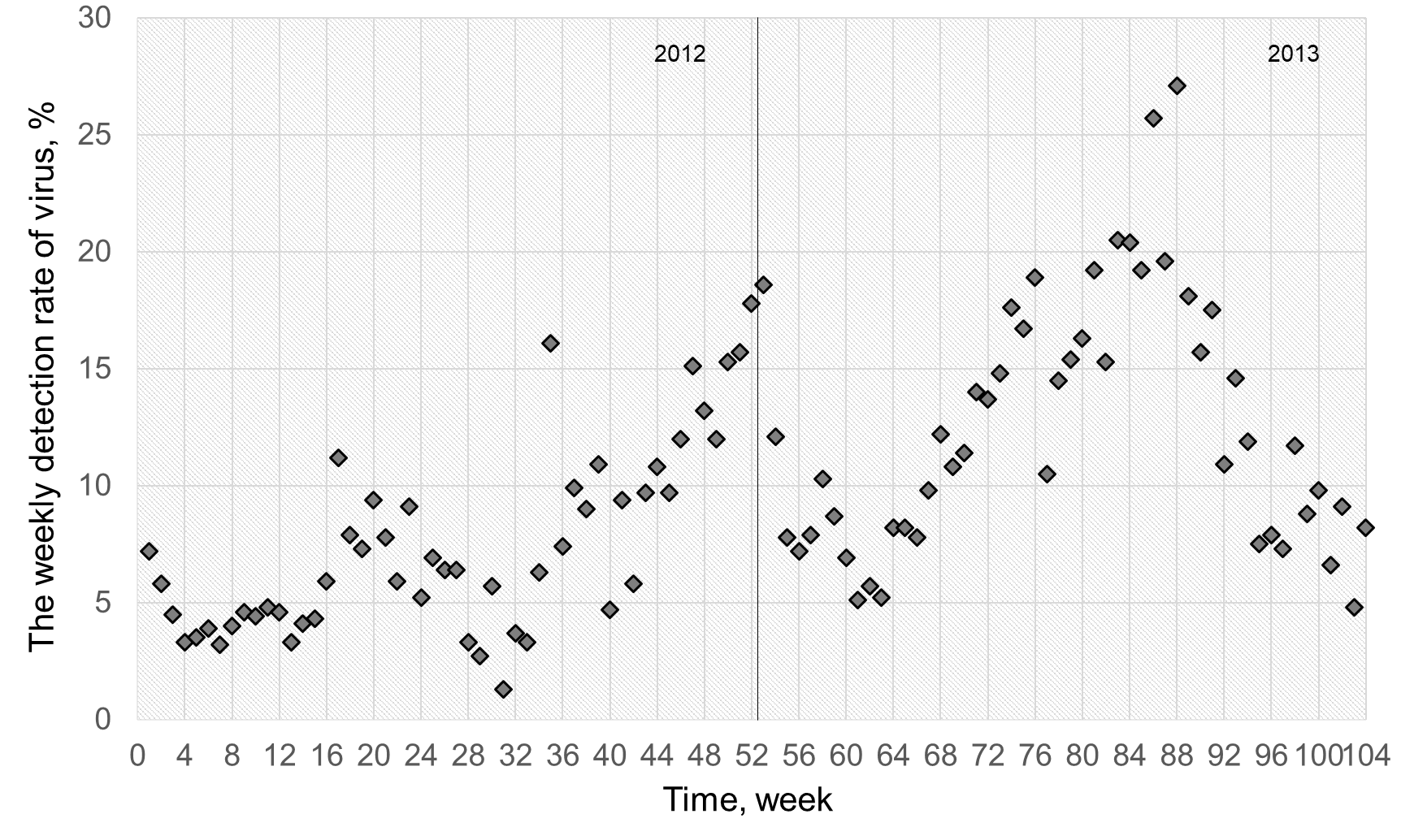


[Parainfluenza virus]


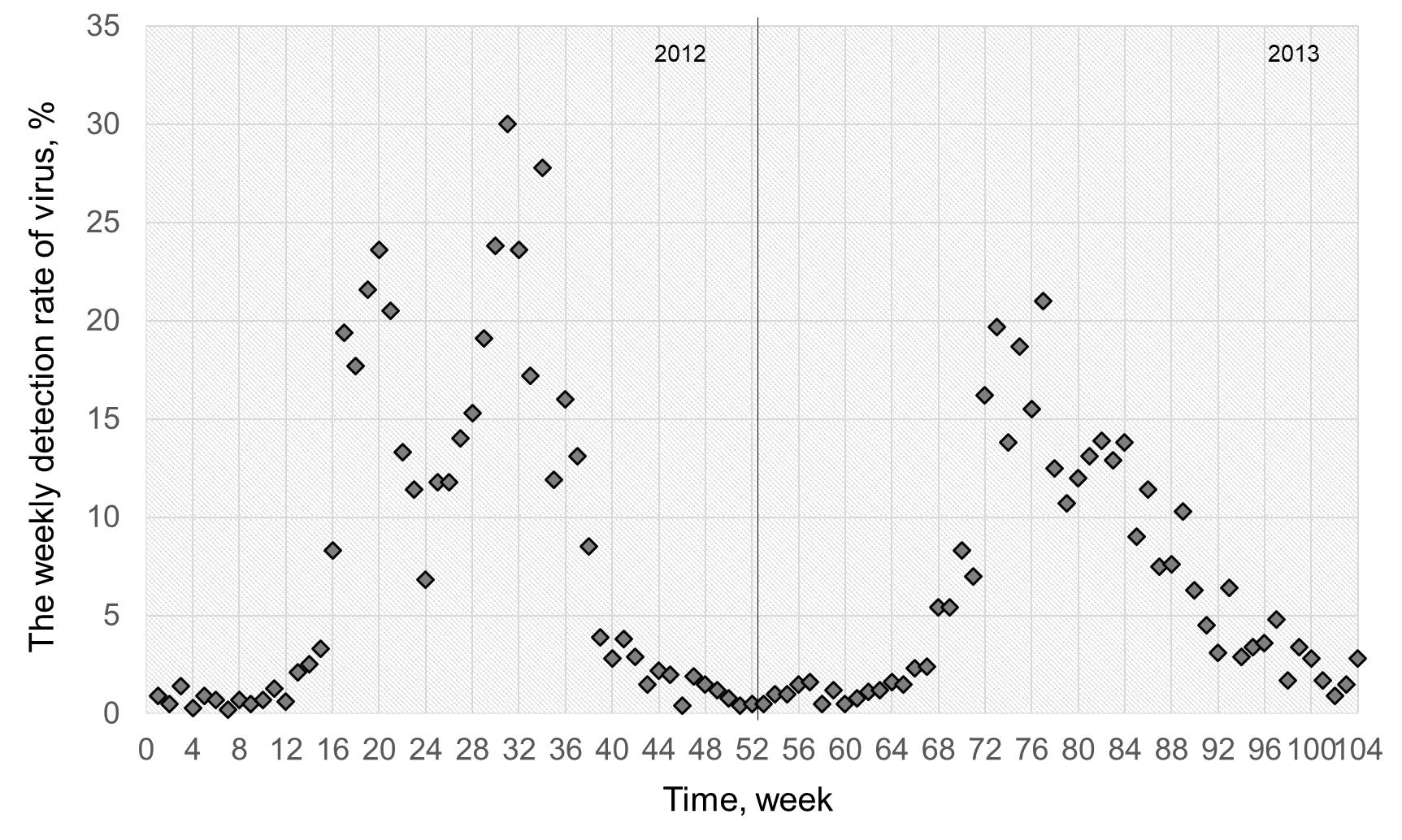


[Respiratory Syncytial virus]


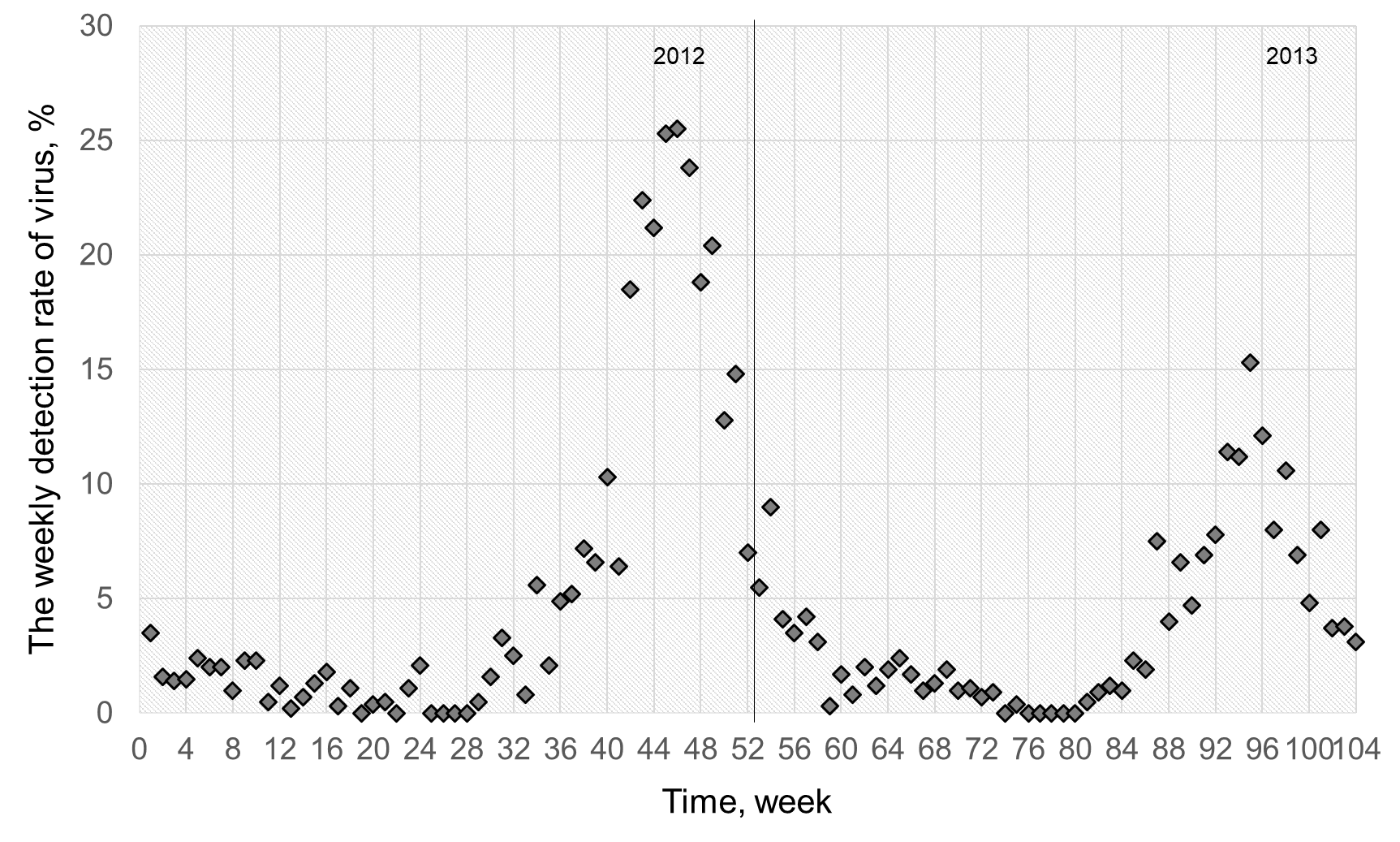


[Influenza virus]


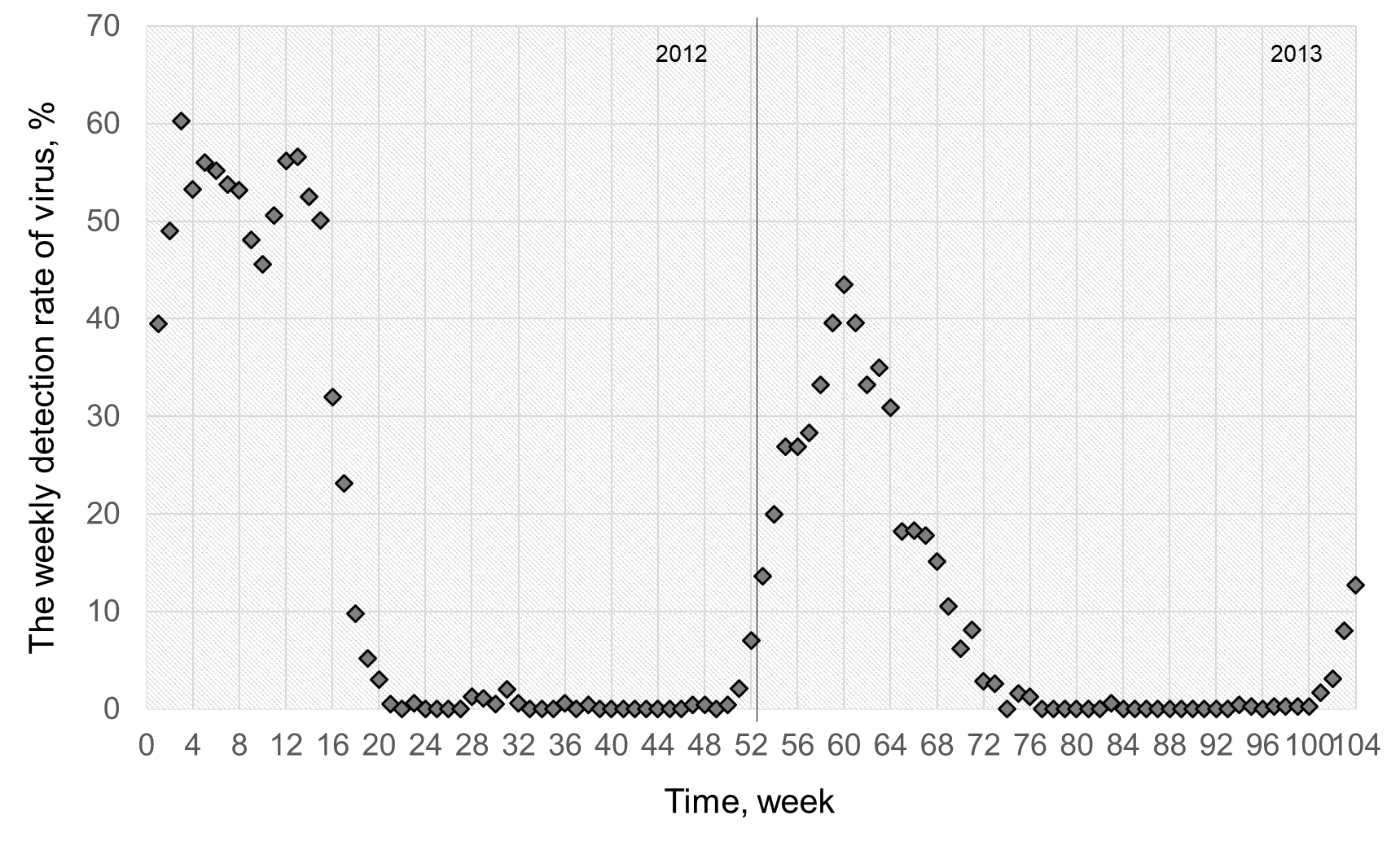


[Coronavirus]


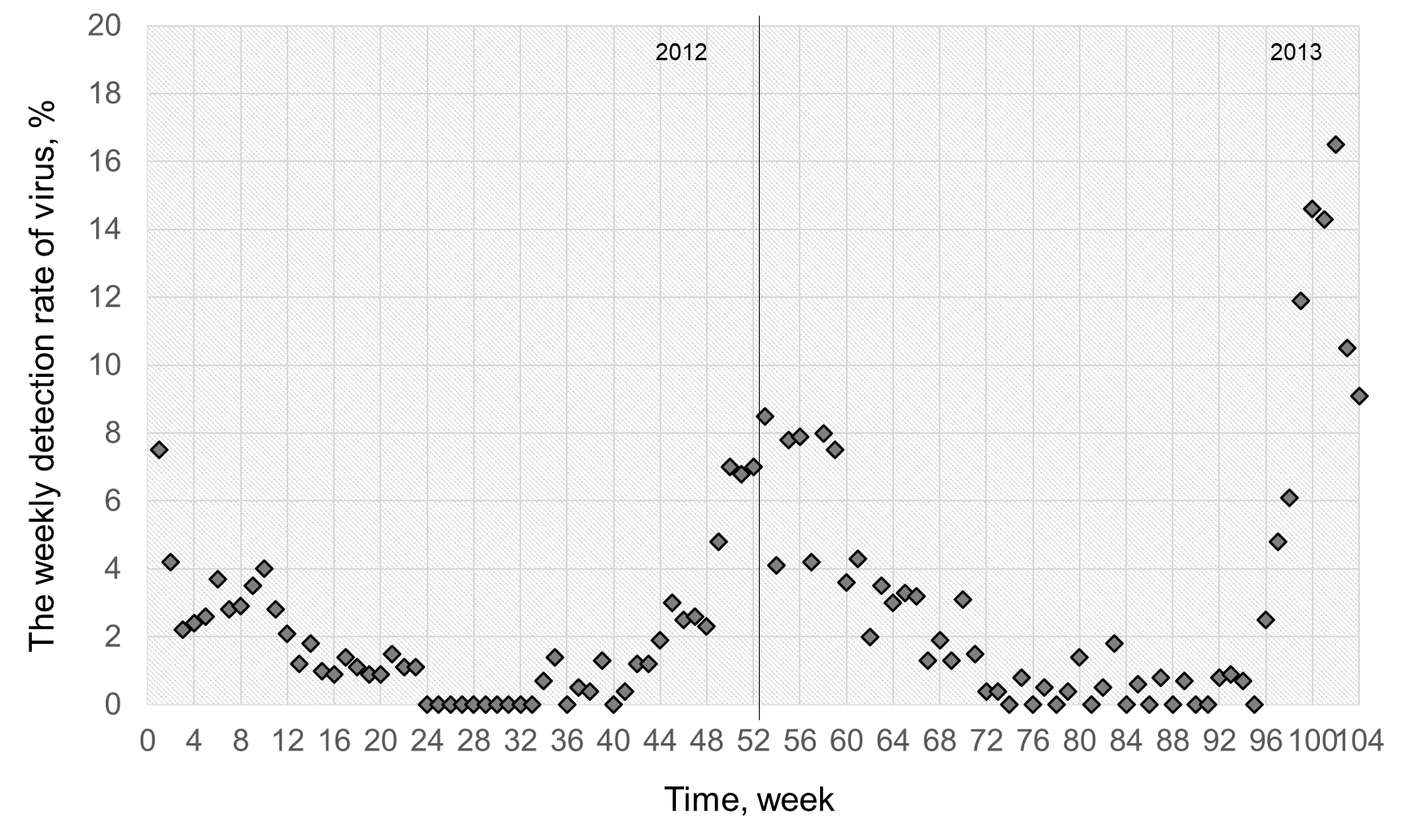


[Rhinovirus]


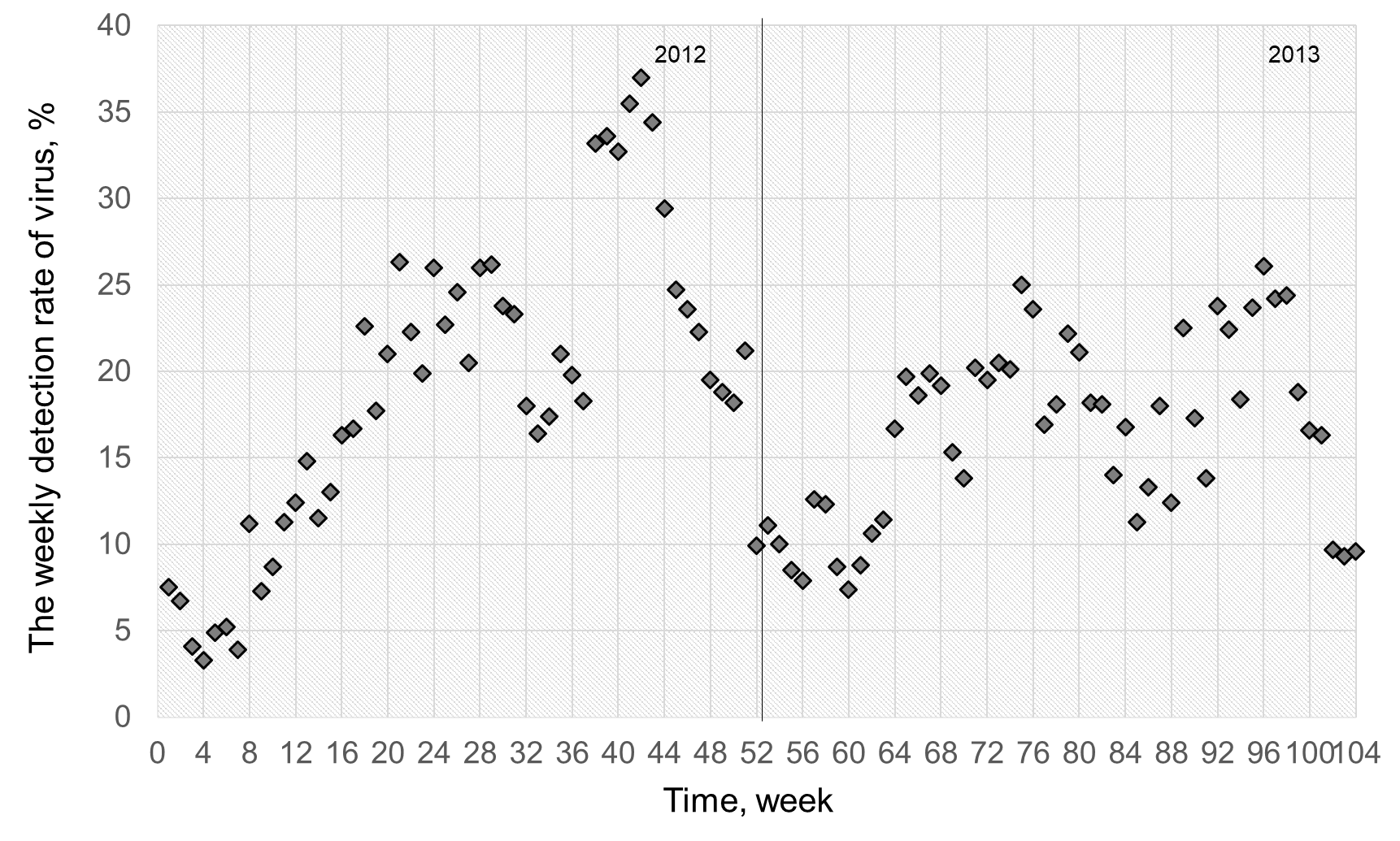


[Bocavirus]


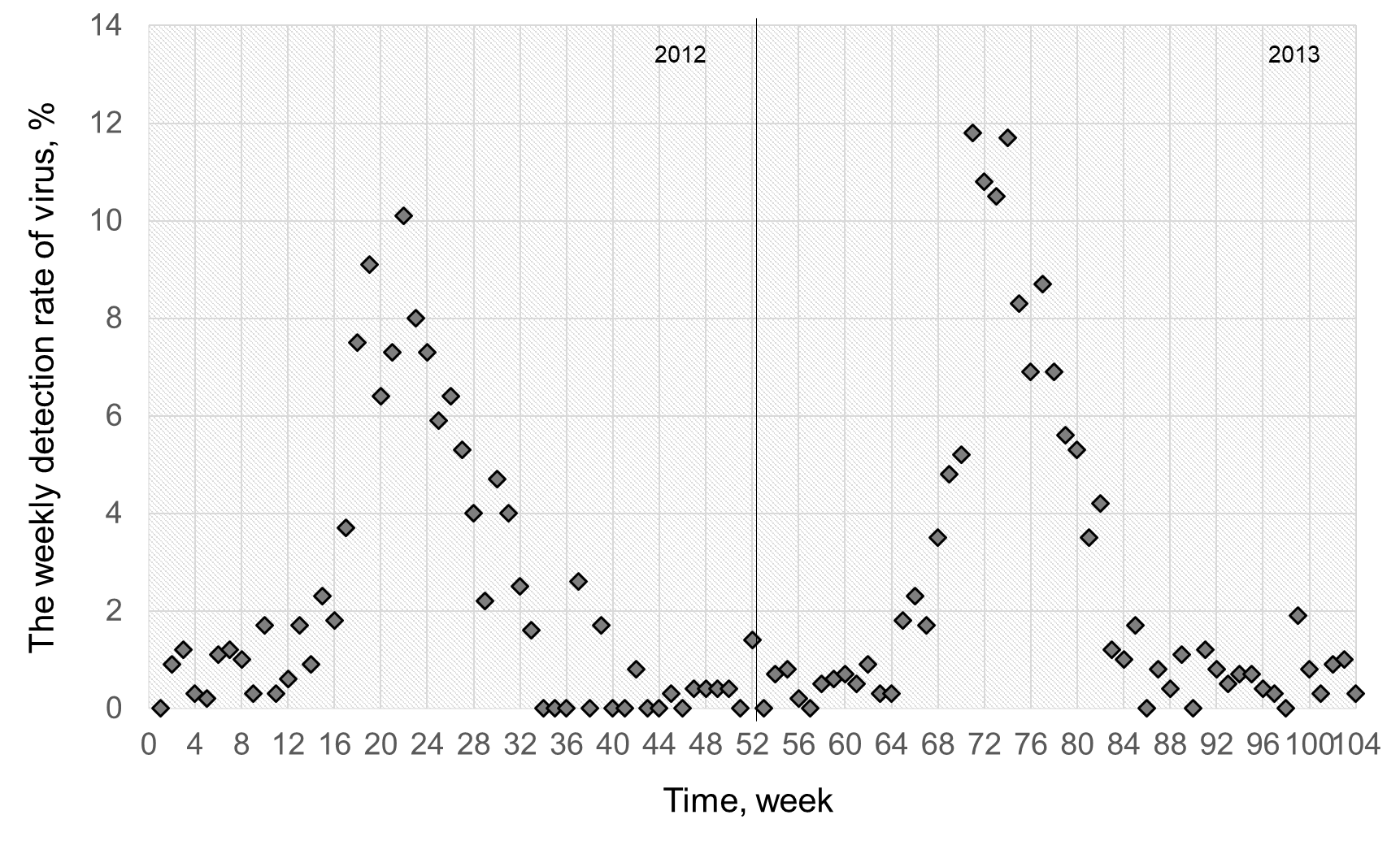


[Metapneumovirus]


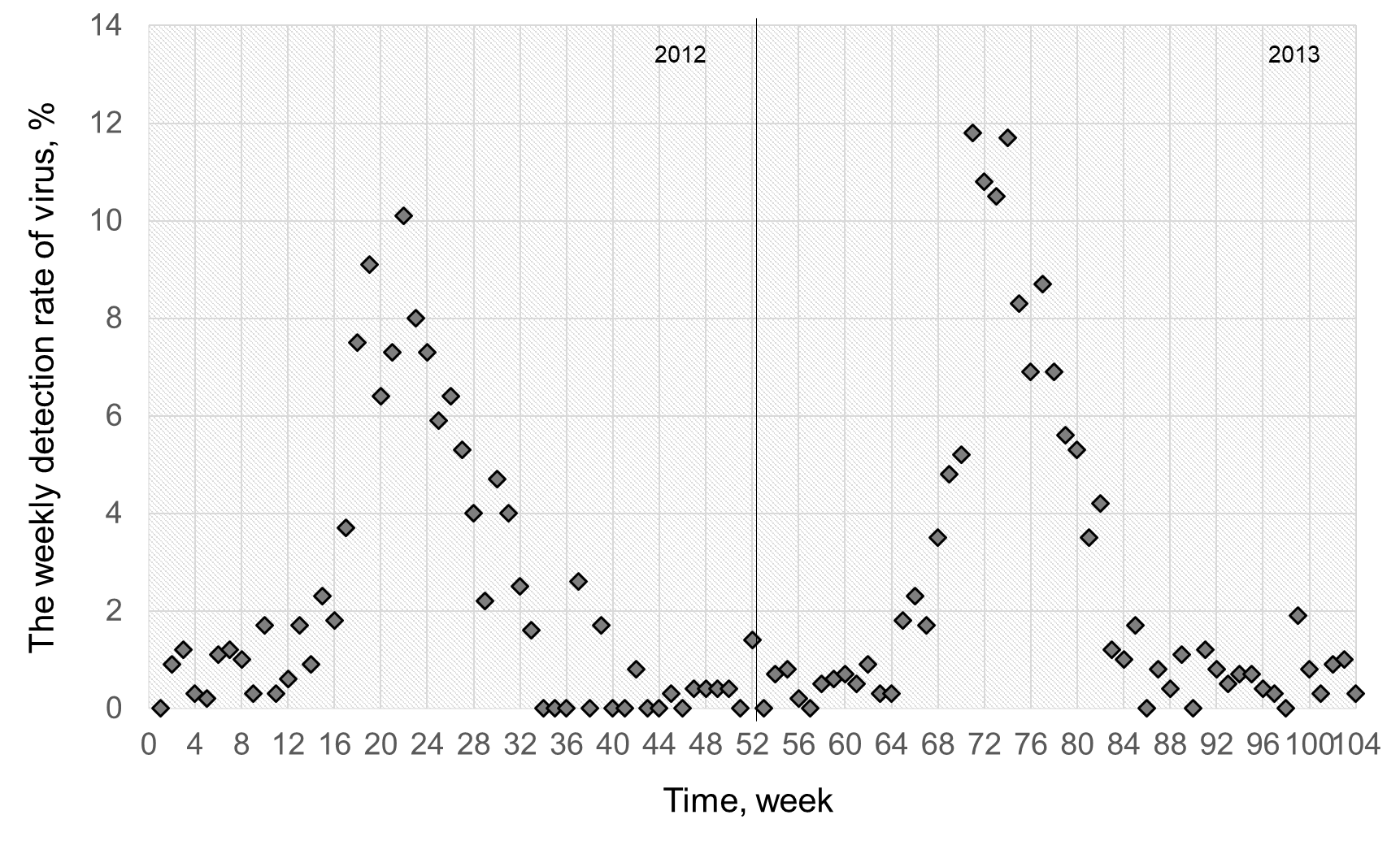


**Figure S2.** The weekly detection rate of eight respiratory virus in 2012 and 2013

The X-axis represents the study period from 2012 to 2013. As the detection rates for respiratory viruses are collected on a weekly basis, week units from the first week of January 2012 to the last week of December 2013 are represented. The Y-axis represents the weekly detection rate for the virus in each week.


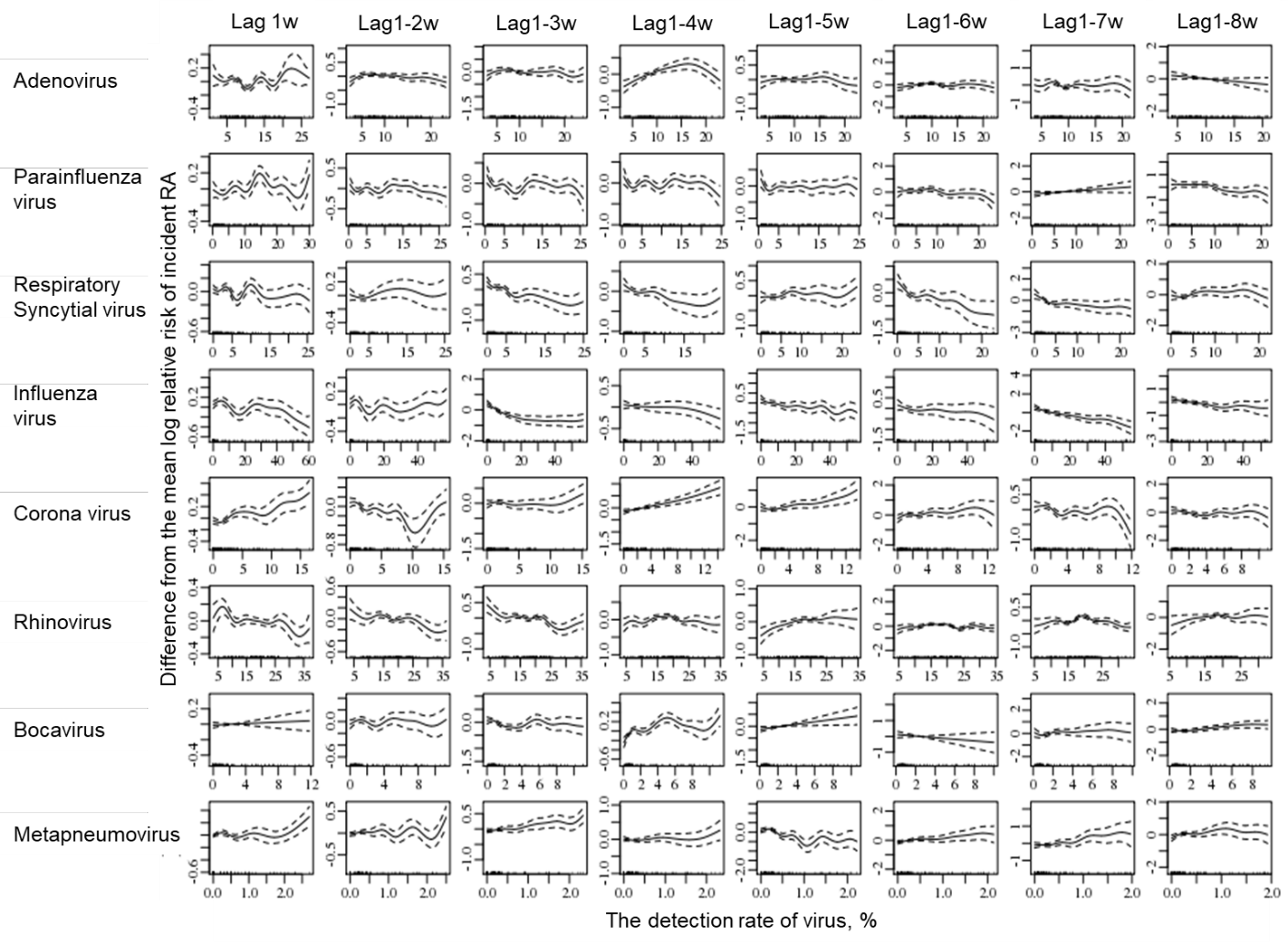


**Figure S3.** Risks of incident RA associated with infection with eight respiratory viruses over 8 lag weeks.

The X- and Y-axes represent the weekly virus detection rate as a percentage and difference from the mean log relative risk of incident RA, respectively. Solid lines represent associations between the weekly virus detection rate and weekly number of incident RA cases and dashed lines represent 95% confidence intervals for the risk.

Each row represents various types of respiratory virus (adenovirus, parainfluenza virus, respiratory syncytial virus, influenza virus, corona virus, rhinovirus, bocavirus, and metapneumovirus).

Each column represents various moving averages for lag weeks of viral infections. For example, ‘Lag1w’ represents the previous one week of viral infections and ‘Lag1-2w’ the moving average of the previous two weeks of viral infection.
